# S100 Calcium Binding Protein A10, A Novel Oncogene, Promotes the Proliferation, Invasion, and Migration of Hepatocellular Carcinoma

**DOI:** 10.3389/fgene.2021.695036

**Published:** 2021-06-11

**Authors:** Xing Zhou, Min Shi, Jun Cao, Tianwen Yuan, Guanzhen Yu, Ying Chen, Wenzheng Fang, Hongwei Li

**Affiliations:** ^1^Department of Interventional Oncology, Dahua Hospital, Shanghai, China; ^2^Department of Pathology, Sichuan Cancer Center, School of Medicine, Sichuan Cancer Hospital & Institute, University of Electronic Science and Technology of China, Chengdu, China; ^3^Department of Oncology, Longhua Hospital Affiliated to Shanghai University of Traditional Chinese Medicine, Shanghai, China; ^4^Shanghai Key Laboratory of Multidimensional Information Processing, East China Normal University, Shanghai, China; ^5^Department of Gastroenterology, Naval Medical University, Shanghai, China; ^6^Department of Oncology, Clinical Medical College of Fujian Medical University (900 Hospital of the Joint Logistics Team), Fujian, China

**Keywords:** S100A10, proliferation, invasion, migration, hepatocellular carcinoma, *in vivo*

## Abstract

Hepatocarcinogenesis is a highly complicated process that is promoted by a series of oncogenes. Our study aims to identify novel oncogenes promoting hepatocellular carcinoma (HCC) by bioinformatic analysis and experimental validation. Here, we reported that S100 calcium binding protein A10 (S100A10) was screened out as a potential novel oncogene in HCC by integrated analysis of OEP000321 dataset and the Cancer Genome Atlas (TCGA)-Liver-Cancer data. Furthermore, S100A10 was highly expressed in HCC samples and observably associated with patients’ overall survival (OS). Overexpression of S100A10 in Hep3B and Huh-7 increased the cell proliferation, whereas downregulation of S100A10 in SK-Hep-1 and HepG2 cells reduced the cell viability to almost stop growing. *In vivo* tumor growth assays showed that S100A10-overexpressing Hep3B cells had a larger tumor size than control. Moreover, S100A10 overexpression promoted Hep3B cells migration and invasion, and S100A10 knockdown inhibited SK-Hep-1 cells migration and invasion, *in vitro*. In conclusion, it is demonstrated that S100A10 is a novel oncogene in HCC, indicating a possible novel therapeutic strategy of HCC.

## Introduction

Hepatocellular carcinoma (HCC) is a malignancy with the highest mortality rate worldwide (Wu et al., 2017). Due to the particular difficulty to diagnose HCC at an early stage, less than 20% of HCC patients have an opportunity to receive curative therapy. Even so, some of these patients still die from tumor recurrence mainly caused by metastasis, resulting in a bad prognosis (Wang et al., 2012; Osaki and Nishikawa, 2015). Therefore, it is of urgency to illustrate the mechanisms of HCC progression and metastasis in order to discover new therapeutic targets.

S100 calcium binding protein A10 (S100A10) belongs to the S100 family of proteins (Tantyo et al., 2019). As an active modulator of various biological functions, it can participate in a variety of protein interactions, including Annexin A2, DLC1, and B-FABP (Hedhli et al., 2012). The abnormal expression of S100A10 affects cell proliferation, apoptosis, angiogenesis, inflammation, and invasion. Numerous studies have confirmed that S100A10 is an oncogene, such as intestinal cancer (Suzuki et al., 2011), basal-type breast cancer (McKiernan et al., 2011), lung cancer (Katono et al., 2016), ovarian cancer (Wang et al., 2019b), pancreatic ductal cancer (Bydoun et al., 2018), and gastric cancer (El-Rifai et al., 2002). S100A10 has also been noted in HCC (Kittaka et al., 2008; Shan et al., 2013; Zhang et al., 2016; Lou et al., 2019). Although, group of Zhou demonstrated that miR-590-5p inhibited S100A10 expression by directly binding to its mRNA 3'UTR, the biological function of S100A10 was not involved (Shan et al., 2013). Herein, the functional role of S100A10 in HCC is currently unclear.

Herein, we initially re-analyzed the data from Fan’s study (Gao et al., 2019) and the Cancer Genome Atlas (TCGA) database, and S100A10 was identified as a novel oncogene promoting hepatocarcinogenesis. Furthermore, a series of functional assays were performed. It was demonstrated that S100A10 promoted the proliferation, invasion, and migration of HCC, indicating a possible novel therapeutic target of HCC.

## Materials and Methods

### Schematic Workflow for Mining of Novel Oncogene in HCC

The expression profile (OEP000321)^1^ was downloaded from Fan’s study (Gao et al., 2019) to screen out the differentially expressed genes (DEGs) and the differentially expressed proteins (DEPs). Combined with TCGA-Liver-Cancer survival data,^2^ the survival-associated DEGs were identified as potential oncogenes for further validation (Supplementary Figure 1).

### Screening of DEGs and DEPs

The raw data from OEP000321 were normalized using the R package “limma” before data mining (Supplementary Figures 2A,B). Then, the normalized data were analyzed to screen the DEGs and DEPs using the limma software package. Value of *p* < 0.05 with | log_2_ (fold change) | > 1 was statistically significant.

### Identification of Survival-Associated Oncogenes

To identify the potential survival-associated oncogenes, we set a strict screening criterion. Briefly, the overlapping genes of DEGs and DEPs were screened out, and the mRNA-protein correlation > 0.45 of overlapping genes was selected for further study. Combined with TCGA survival data, the selected genes were separated into two groups based on *p*-value. Only the survival-associated (value of *p* < 0.05) selected genes were identified as candidate oncogenes.

### Cell Lines and Cell Culture

Hepatocellular carcinoma cell lines Hep3B (HB-8064), Huh-7 (PTA-4583), SK-Hep-1 (HTB-52), and HepG2 (HB-8065) were bought from the American Tissue Culture Collection (ATCC). DMEM (Thermo Fisher Scientific, MA, United States) with 10% FBS (Thermo Fisher Scientific, MA, United States) was used to culture all the cell lines in a humidified incubator containing 5% CO_2_ at 37°C.

### Construction of Plasmids, Transfection and Establishment of Stable Cell Line

The full-length cDNA of S100A10 obtained from YFP-human S100A10 expression plasmid (Addgene, #107200) was inserted into pcDNA3.1 to generate pcDNA3.1-S100A10. The siRNA sequence targeting S100A10 was purchased from Merck (EHU046811, Darmstadt, Germany). Transfection assay utilized Lipofectamine 3000 (Invitrogen, United States). To establish a stable S100A10 overexpression cell line for *in vivo* tumor growth assay, Hep3B cells were transfected with pcDNA3.1-S100A10 or pcDNA3.1 vector, respectively. About 48 h later, the supernatant was substituted with the fresh medium containing G418. Around 2 weeks later, the survival single clone was digested and re-seeded in 35-mm dishes. The siRNA sequences were listed as follows: Si-S100A10, 5'-GUGGGCUUCCAGAGCUUCU-3'; Si-Control, 5'-GCAGAAGGGAAAGAAGUAG-3'.

### Real-Time Quantitative PCR

Total RNA got extraction using TRIzol reagent (Invitrogen, United States). Real-time quantitative PCR (RT-qPCR) applied SYBR® Premix Ex Taq Kit (TAKARA). Next, the 2^-ΔΔCt^ method was employed to access the relative mRNA expression. The primers were listed as follows: S100A10-F: 5'-CACACCTTGATGCGTCCTCT-3' and S100A10-R: 5'-GGCAACCGGATGCAAACAAT-3'; β-actin-F: 5'-CTCCATCCTGGCCTCGCTGT-3' and β-actin-R: 5'-GCTGTCACCTTCACCGTTCC-3'.

### Western Blotting

Cell lysates were obtained using RIPA buffer (Beyotime, China) and supplemented with loading buffer. The secreted protein in the conditioned medium was collected by ethanol precipitation. In short, 95% ethanol was added to the conditioned media and kept at 20°C overnight. The precipitated protein was collected with SDS loading buffer, and a standard western blot was performed immediately. Western blotting was performed using SDS-PAGE gel for protein separation and nitrocellulose membrane (Millipore, United States) for western blotting. The membrane was blocked with 5% nonfat milk (BD Biosciences, United States) in Tris buffered saline (TBS), and incubated with primary antibodies diluted in TBS containing 1% bovine serum albumin at 4°C overnight. The Odyssey imaging system (LI-COR Biosciences, United States) was used to detect the bound antibody, and the secondary antibody was labeled with DyLight fluorescent dye. The primary antibodies were anti-S100A10 (1:2,500, GTX100697, GeneTex) and anti-β-actin (1:5,000, A5316, Sigma).

### Cell Proliferation Assay

Cells were seeded into 96-well plates at a density of 1 × 10^3^ cells per well in triplicate. Cell Counting kit 8 (CCK-8, Dojindo, Kumamoto, Japan) was used to estimate cell growth. The specific operation was as the manufacturer’s instructions. The absorbance was selected at 450 nm using a spectrophotometer (Bio-Rad, CA).

### *In vivo* Tumor Growth Assays

NOD/SCID mice (6 weeks) got bought from the Animal Center of Shanghai (Shanghai SLAC Laboratory Animal, China). All experimental procedures were performed according to the Institutional Animal Ethical Committee of Longhua Hospital. Stable S100A10 overexpression and the negative control (pcDNA3.1) Hep3B cells (5 × 10^6^ cells/animal) were injected subcutaneously into nude mice (four mice/group) to produce implanted tumors. Tumor volumes were calculated as follows: volume = (the larger diameter) × (the smaller diameter)^2^/2 (Qin et al., 2019). Mice were sacrificed by CO_2_ asphyxiation 54 days later. Tumor weight and volume were measured, respectively.

### Cell Migration and Invasion Assays

For migration assays, HCC cells were plated on membranes with 8.0 μm pores. For invasion assays, HCC cells were planted in Matrigel-coated chambers (Corning Incorporated, Corning, NY). Adhered cells were fixed and stained with 0.1% crystal violet for 1 min.

### Statistical Analysis

Statistical analyses were performed with Graphpad 8.2.1 (San Diego, United States). Data from three independent experiments were expressed as the mean ± SD. We used chi-square test or Fisher’s exact test to dissect categorical data. Student *t*-tests were used to evaluate comparisons between the two groups. Survival curves were made a comparison by a log-rank test. Value of *p* less than 0.05 was seen as statistically significant.

## Results

### S100A10 Is Identified as a Potential Oncogene in HCC

**Figure 1:**
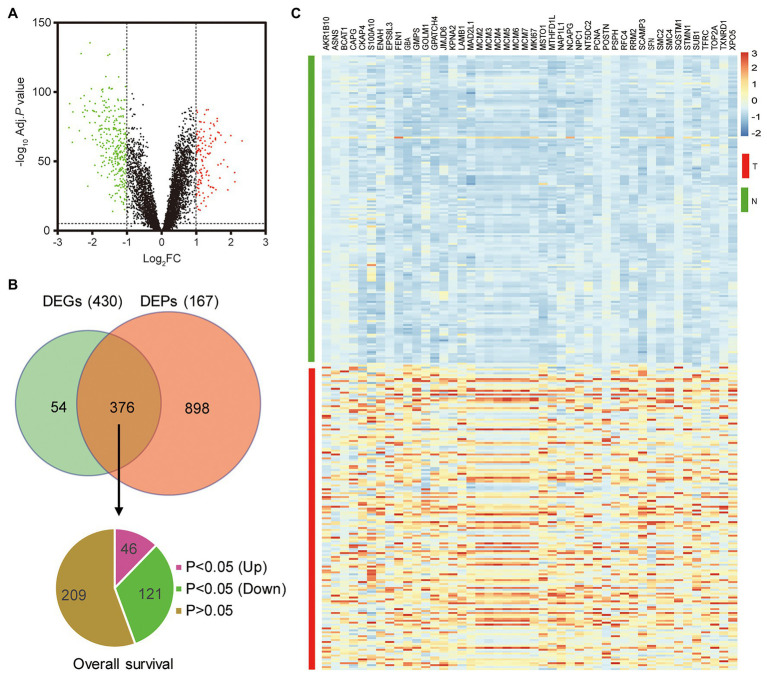
S100 calcium binding protein A10 (S100A10) is identified as a potential oncogene in hepatocellular carcinoma (HCC). **(A)** Volcano plot of the differentially expressed genes (DEGs). The red dots were increasing genes, and green dots were reducing genes in HCC tumor samples, respectively. **(B)** Identification of potential oncogene in HCC. Venn diagram of DEGs and differentially expressed proteins (DEPs) with miRNA-protein correlation > 0.45. Pie chart of 376 selected genes, including 167 (46 upregulated and 121 downregulated in HCC) survival-associated genes and 209 non-survival-associated genes. **(C)** Heat map of the 46 potential oncogenes.

To explore the novel oncogenes in HCC, we re-analyzed the gene expression profile and the protein expression profile (OEP000321) obtained from Fan’s study (Gao et al., 2019). A total of 430 DEGs and 1,271 DEPs were identified between HCC tumor samples and non-tumor samples (Figure 1A). Furthermore, the 376 overlapping genes with mRNA-protein correlation > 0.45 were selected as candidate genes (Figure 1B). Combined with TCGA survival data, 167 survival-associated genes (*p* < 0.05) were screened out, including 46 upregulated genes, which were regarded as potential oncogenes in HCC (Figure 1C). Among the 46 selected genes, many genes have been previously reported as oncogenes in HCC, such as AKR1B10 (Ye et al., 2019), ASNS (Zhang et al., 2013), BCAT1 (Ji et al., 2019), and so on. Moreover, we found a S100 protein family member, S100A10 was identified in our analysis. Currently, there are only four studies that noted S100A10 in HCC, none of which report the functional role of S100A10 in HCC.

### S100A10 Is Overexpressed in HCC and Correlated With Poor Prognosis

**Figure 2:**
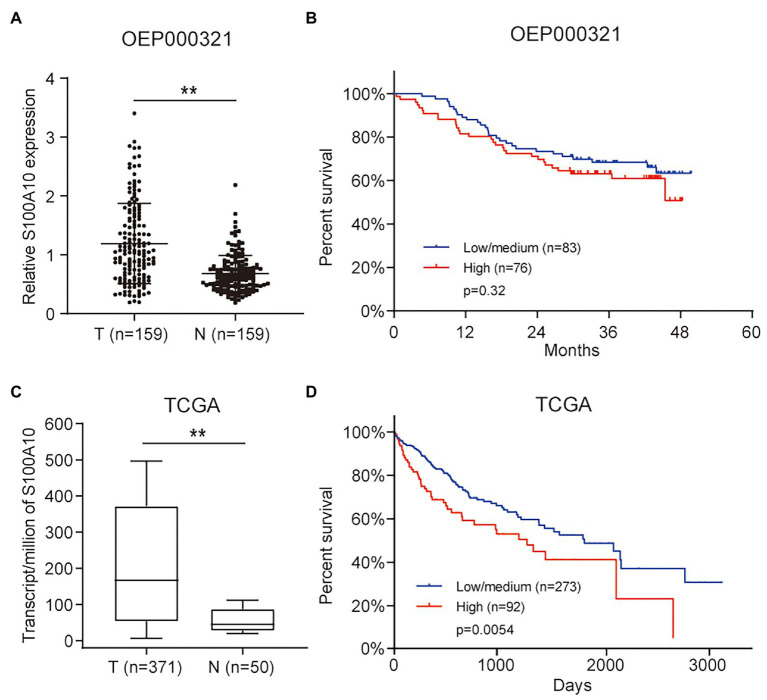
S100 calcium binding protein A10 is upregulated in HCC and correlated with poor prognosis. **(A)** S100A10 expression in HCC tissues and adjacent normal tissues. **(B)** Correlations between S100A10 expression and the overall survival (OS) of HCC patients. **(C)** S100A10 expression based on the Cancer Genome Atlas (TCGA) cohort. **(D)** Correlations between S100A10 expression and the OS based on TCGA cohort. ^**^p < 0.01.

Based on the analytic results, we analyzed S100A10 data in HCC using OEP000321. It was demonstrated that the expression of S100A10 was significantly higher in HCC samples, but was not significantly associated with overall survival (OS; Figures 2A,B). At the gene expression level, a similar result was obtained by analyzing HCC data from TCGA (Figure 2C). Unlike the OS results in OEP000321, high expressions of S100A10 were significantly associated with worse OS in TCGA (Figure 2D). These results above imply that S100A10 is an oncogene in HCC.

### S100A10 Advances HCC Growth *in vitro* and *in vivo*

**Figure 3:**
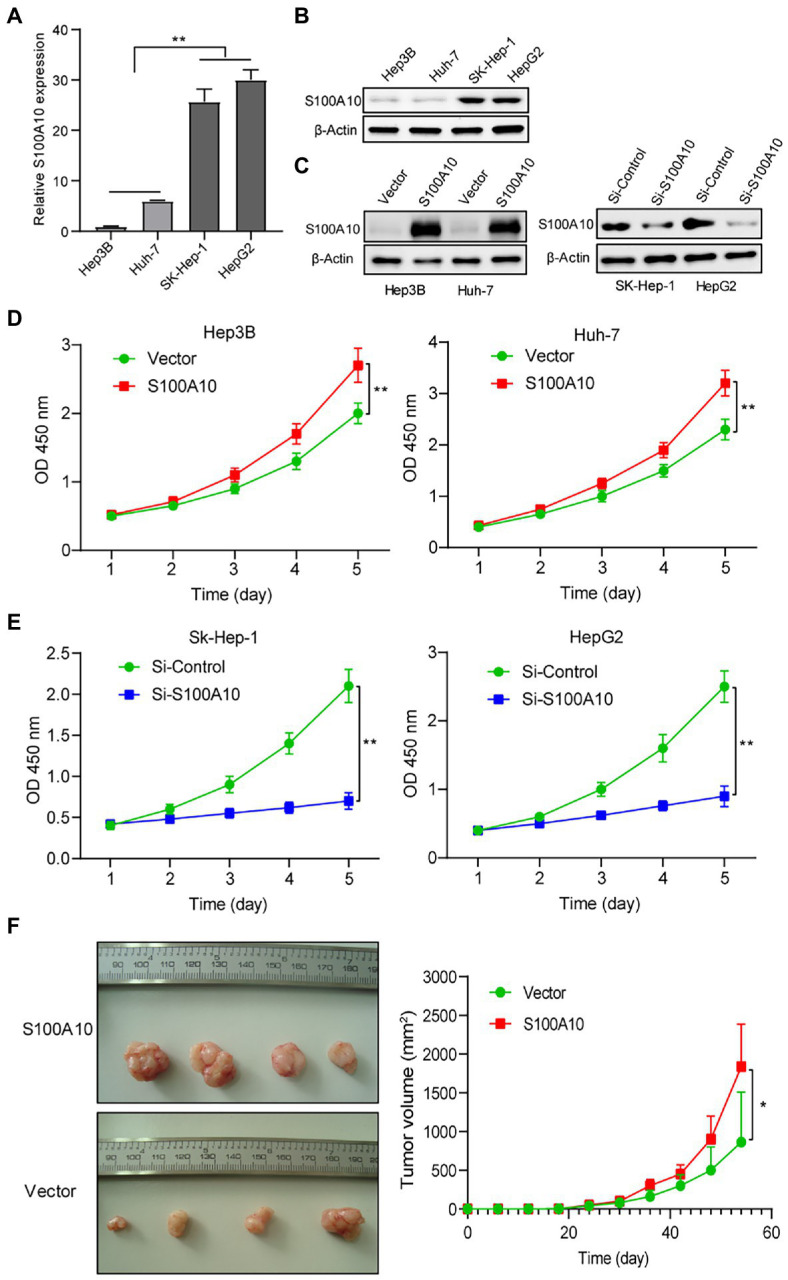
S100 calcium binding protein A10 advances HCC growth *in vitro* and *in vivo*. **(A)** The mRNA level of S100A10 in HCC cell lines (Hep3B, Huh-7, SK-Hep-1, and HepG2). **(B)** The protein level of S100A10 in HCC cell lines. **(C)** The level of S100A10 in treated HCC cell lines were accessed by western blotting. **(D)** Cell Counting kit 8 (CCK-8) indicated overexpressing S100A10 enhanced the proliferation of Hep3B, Huh-7 cells. **(E)** Knocking down S100A10 blocked SK-Hep-1 and HepG2 cell growth. **(F)** Overexpressing S100A10 enhanced the subcutaneous growth of Huh-7 cells in nude mice. ^*^p < 0.05; ^**^p < 0.01.

To verify our findings above, we first detected the expression of S100A10 in SK-Hep-1, Hep3B, Huh-7, and HepG2 cell lines at mRNA and protein levels. We found that S100A10 expressed at a low level in Hep3B and Huh-7, while the expressions of S100A10 in SK-Hep-1 and HepG2 were higher (Figures 3A,B). Overexpression of S100A10 assays was performed in Hep3B and Huh-7. Meanwhile, knockdown of S100A10 assays was performed in SK-Hep-1 and HepG2 (Figure 3C). After overexpressing S100A10, Hep3B, and Huh-7 cell proliferation was increased (Figure 3D), whereas knockdown of S100A10 suppressed SK-Hep-1 and HepG2 cell proliferation (Figure 3E). Of note, we found that SK-Hep-1 and HepG2 almost stopped growing when S100A10 was knocked down (Figure 3E). In addition to *in vitro* experiments, we also examined the influence of S100A10 on tumorigenesis *in vivo*. Our preliminary experimental results showed that the S100A10-knockdown SK-Hep-1 and HepG2 cells failed in tumorigenesis, due to the extremely low growth rate. The stable S100A10-overexpressing Hep3B cells and vector control Hep3B cells got injected to form subcutaneous tumors in nude mice. The data proved S100A10 expression was positively related to subcutaneous tumor size (Figure 3F). Collectively, it is demonstrated that S100A10 promotes HCC growth *in vitro* and *in vivo*.

### S100A10 Promoted HCC Migration and Invasion

**Figure 4:**
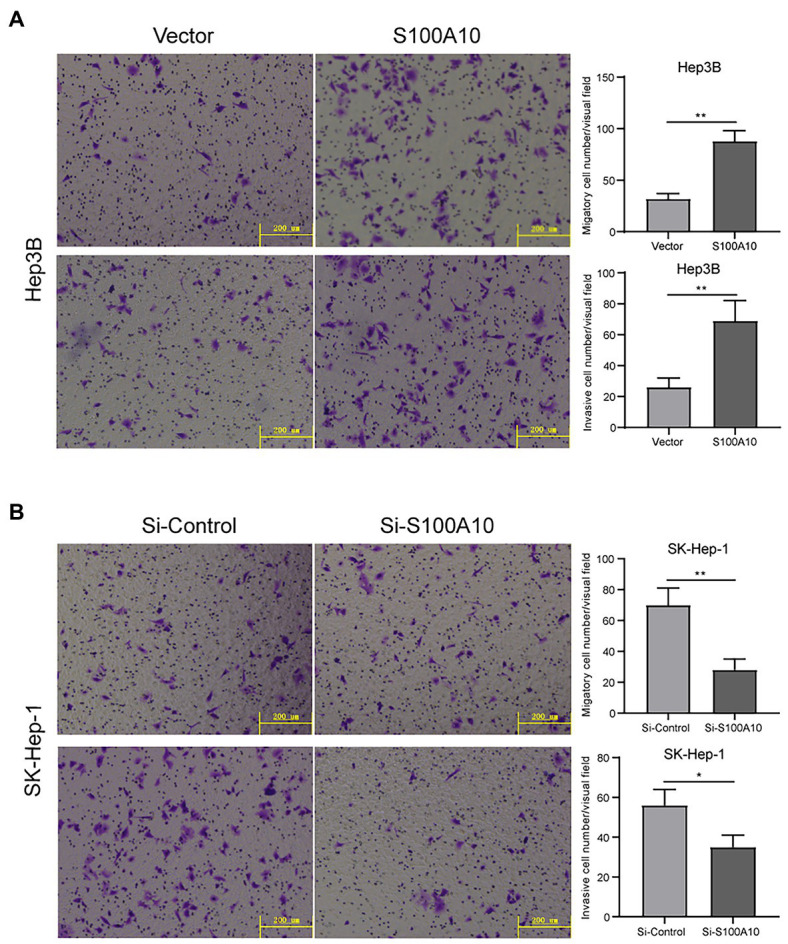
S100 calcium binding protein A10 promoted cell migration and invasion. **(A)** Overexpressing S100A10 enhanced the migration and invasion of Hep3B cells. **(B)** Knocked S100A10 reduced the migration and invasion of SK-Hep-1 cells. ^*^p < 0.05; ^**^p < 0.01.

Due to the correlation between S100A10 expression and OS, we hypothesized that S100A10 played a critical role in HCC metastasis. Transwell assays showed that S100A10 overexpression could increase Hep3B cell migration and invasion (Figure 4A). On the contrary, when the expression of S100A10 was knocked down in SK-Hep-1 cells, the migration and invasion were reduced (Figure 4B). These results illustrate that S100A10 works as a pro-metastasis protein in HCC. Currently, the mechanism underlying the pro-metastatic ability of Sl00A10 is unknown. We tend toward the explanation that S100A10 HCC metastasis directly affecting MMP regulation to influence plasmin generation (Miller et al., 2017).

## Discussion

Hepatocellular carcinoma has been considered as the sixth commonest solid tumor and the third most lethal malignancy worldwide (Osaki and Nishikawa, 2015). Recently, with the improvement of early diagnosis, effective monitoring treatments, the OS of HCC patients has increased (Kudo, 2017). However, the long-term survival rate is still unsatisfactory because of the death caused by recurrence and metastasis (Zhu et al., 2011). So, it is imperative to uncover the novel pathogenic mechanism, particularly to identify novel oncogenes for a better understanding of this disease.

S100 protein is a Ca^2+^ binding regulatory protein that is involved in cell cycle progression, cell differentiation, and cytoskeleton membrane interactions (Bresnick, 2018). Previous studies have reported the aberrant expression of S100 proteins in cancer (Tong et al., 2014; Brenner and Bruserud, 2018) and immune-related disease (Manolakis et al., 2011; Yammani, 2012). As a S100 protein (Bydoun and Waisman, 2014; Holthenrich and Gerke, 2018), S100A10 has increased expressions in breast cancer (McKiernan et al., 2011), lung cancer (Katono et al., 2016), gastric cancer (El-Rifai et al., 2002), and pancreatic ductal cancer (Bydoun et al., 2018).

Nevertheless, the expression of S100A10 and its biological functions have not been elucidated in HCC. As the only research of S100A10 in HCC, Zhou reported that miR-590-5p could directly bind the 3'-UTR of S100A10 mRNA to reduce its expression, thereby inhibiting the growth of HepG2 cells (Shan et al., 2013). Unfortunately, this study just demonstrated the targeting of S100A10 by miR-590-5p, without the biological function of S100A10. To make up the functional blank, we performed a series of analysis and validation experiments and reported a comprehensive study on S100A10 in HCC for the first time. Herein, we found that the levels of S100A10 were substantially higher in HCC samples than that in normal samples. Additionally, we also found that the expression of S100A10 was negatively associated with the OS in TCGA survival data. Strangely, this result is not consistent with the result obtained from Fan’s data (Figures 2A,B). This might be due to the incomplete follow-up data. Furthermore, we explored the role of S100A10 in HCC cell growth.

Previous studies reported that S100A10 accumulation was involved in gastric cancer cell invasion and migration through the succinyltransferase CPT1A and SIRT5-mediated desuccinylation (Wang et al., 2019a). Additionally, S100A10 also regulated the metastasis of lung cancer cells by the interaction with DLC1 (Yang et al., 2011). Recently, Cui’s group reported that S100A10 was an oncogene in ovarian cancer by promoting tumor metastasis, and reduced sensitivity to carboplatin (Wang et al., 2019b). In our study, we found that S100A10 promoted the migration and invasion of Hep3B cells. When S100A10 got knocked down in SK-Hep-1 cells, the migrating and invading were reduced.

This study has some limitations. First, we should detect the expression level of S100A10 in normal liver cell lines, and further evaluate the expression level of S100A10 in HCC. Second, we need to collect clinical samples to explore the correlation between S100A10 expression and clinical parameters (including clinical stage, age, and survival time). Finally, the mechanisms in the cancer-promoting activity of S100A10 are still unknown. Therefore, further studies are required for this novel oncogene in HCC.

To sum up, to our knowledge, we reported for the first time the functional role of S100A10 in HCC. This research indicates the relevance between S100A10 and HCC patient’s OS. A series of functional analyses showed that S100A10 promoted the proliferation, invasion, and migration of HCC, indicating that it may be a new therapeutic target for HCC. This research emphasizes the oncogene role of S100A10, deepening our understanding of S100A10 in hepatocarcinogenesis.

## Data Availability Statement

The original contributions presented in the study are included in the article/Supplementary Material, further inquiries can be directed to the corresponding authors.

## Ethics Statement

All experimental procedures were performed according to the Institutional Animal Ethical Committee of Longhua Hospital.

## Author Contributions

XZ, MS, WF, and HL performed the study and drafted the article. JC, TY, GY, and YC conducted data acquisition, data analysis, and interpretation. All authors discussed the results and agreed to be accountable for all aspects of the work. All authors contributed to the article and approved the submitted version.

### Conflict of Interest

The authors declare that the research was conducted in the absence of any commercial or financial relationships that could be construed as a potential conflict of interest.

## Footnotes

^1^https://www.biosino.org/node/project/detail/OEP000321

^2^https://www.cancer.gov/about-nci/organization/ccg/research/structural-genomics/tcga

## Supplementary Material

Supplementary Figure 1 The survival-associated DEGs were identified as potential oncogenes for further validation.

Supplementary Figure 2 The raw data from OEP000321 were normalized using the R package “limma.”

## References

[ref1] Brenner A. K. Bruserud O. (2018). S100 proteins in acute myeloid leukemia. Neoplasia 20, 1175–1186. 10.1016/j.neo.2018.09.007, PMID: 30366122 PMC6215056

[ref2] Bresnick A. R. (2018). S100 proteins as therapeutic targets. Biophys. Rev. 10, 1617–1629. 10.1007/s12551-018-0471-y, PMID: 30382555 PMC6297089

[ref3] Bydoun M. Sterea A. Liptay H. Uzans A. Huang W. Y. Rodrigues G. J.. (2018). S100A10, a novel biomarker in pancreatic ductal adenocarcinoma. Mol. Oncol. 12, 1895–1916. 10.1002/1878-0261.12356, PMID: 30009399 PMC6210040

[ref4] Bydoun M. Waisman D. M. (2014). On the contribution of S100A10 and annexin A2 to plasminogen activation and oncogenesis: an enduring ambiguity. Future Oncol. 10, 2469–2479. 10.2217/fon.14.163, PMID: 25525855

[ref5] El-Rifai W. Moskaluk C. A. Abdrabbo M. K. Harper J. Yoshida C. Riggins G. J.. (2002). Gastric cancers overexpress S100A calcium-binding proteins. Cancer Res. 62, 6823–6826. PMID: 12460893

[ref6] Gao Q. Zhu H. Dong L. Shi W. Chen R. Song Z.. (2019). Integrated proteogenomic characterization of HBV-related hepatocellular carcinoma. Cell 179, 561.e22–577.e22. 10.1016/j.cell.2019.08.052, PMID: 31585088

[ref7] Hedhli N. Falcone D. J. Huang B. Cesarman-Maus G. Kraemer R. Zhai H.. (2012). The annexin A2/S100A10 system in health and disease: emerging paradigms. J. Biomed. Biotechnol. 2012:406273. 10.1155/2012/406273, PMID: 23193360 PMC3496855

[ref8] Holthenrich A. Gerke V. (2018). Regulation of von-willebrand factor secretion from endothelial cells by the annexin A2-S100A10 complex. Int. J. Mol. Sci. 19:1752. 10.3390/ijms19061752, PMID: 29899263 PMC6032327

[ref9] Ji D. Jiang C. Zhang L. Liang N. Jiang T. Yang B.. (2019). LncRNA CRNDE promotes hepatocellular carcinoma cell proliferation, invasion, and migration through regulating miR-203/BCAT1 axis. J. Cell. Physiol. 234, 6548–6560. 10.1002/jcp.27396, PMID: 30230527

[ref10] Katono K. Sato Y. Jiang S. X. Kobayashi M. Saito K. Nagashio R.. (2016). Clinicopathological significance of S100A10 expression in lung adenocarcinomas. Asian Pac. J. Cancer Prev. 17, 289–294. 10.7314/APJCP.2016.17.1.289, PMID: 26838226

[ref11] Kittaka N. Takemasa I. Takeda Y. Marubashi S. Nagano H. Umeshita K.. (2008). Molecular mapping of human hepatocellular carcinoma provides deeper biological insight from genomic data. Eur. J. Cancer 44, 885–897. 10.1016/j.ejca.2008.02.019, PMID: 18337085

[ref12] Kudo M. (2017). Systemic therapy for hepatocellular carcinoma: 2017 update. Oncology 93, 135–146. 10.1159/000481244, PMID: 29258077

[ref13] Lou Y. Yu Y. Xu X. Zhou S. Shen H. Fan T.. (2019). Long non-coding RNA LUCAT1 promotes tumourigenesis by inhibiting ANXA2 phosphorylation in hepatocellular carcinoma. J. Cell. Mol. Med. 23, 1873–1884. 10.1111/jcmm.14088, PMID: 30588744 PMC6378214

[ref14] Manolakis A. C. Kapsoritakis A. N. Tiaka E. K. Potamianos S. P. (2011). Calprotectin, calgranulin C, and other members of the s100 protein family in inflammatory bowel disease. Dig. Dis. Sci. 56, 1601–1611. 10.1007/s10620-010-1494-9, PMID: 21203903

[ref15] McKiernan E. McDermott E. W. Evoy D. Crown J. Duffy M. J. (2011). The role of S100 genes in breast cancer progression. Tumour Biol. 32, 441–450. 10.1007/s13277-010-0137-2, PMID: 21153724

[ref16] Miller V. A. Madureira P. A. Kamaludin A. A. Komar J. Sharma V. Sahni G.. (2017). Mechanism of plasmin generation by S100A10. Thromb. Haemost. 117, 1058–1071. 10.1160/TH16-12-0936, PMID: 28382372

[ref17] Osaki Y. Nishikawa H. (2015). Treatment for hepatocellular carcinoma in Japan over the last three decades: our experience and published work review. Hepatol. Res. 45, 59–74. 10.1111/hepr.12378, PMID: 24965914 PMC4313689

[ref18] Qin Z. Xiang C. Zhong F. Liu Y. Dong Q. Li K.. (2019). Transketolase (TKT) activity and nuclear localization promote hepatocellular carcinoma in a metabolic and a non-metabolic manner. J. Exp. Clin. Cancer Res. 38:154. 10.1186/s13046-019-1131-1, PMID: 30971297 PMC6458711

[ref19] Shan X. Miao Y. Fan R. Qian H. Chen P. Liu H.. (2013). MiR-590-5P inhibits growth of HepG2 cells via decrease of S100A10 expression and inhibition of the Wnt pathway. Int. J. Mol. Sci. 14, 8556–8569. 10.3390/ijms14048556, PMID: 23598417 PMC3645761

[ref20] Suzuki S. Yamayoshi Y. Nishimuta A. Tanigawara Y. (2011). S100A10 protein expression is associated with oxaliplatin sensitivity in human colorectal cancer cells. Proteome Sci. 9:76. 10.1186/1477-5956-9-76, PMID: 22206547 PMC3317844

[ref21] Tantyo N. A. Karyadi A. S. Rasman S. Z. Salim M. R. G. Devina A. Sumarpo A. (2019). The prognostic value of S100A10 expression in cancer. Oncol. Lett. 17, 1417–1424. 10.3892/ol.2018.9751, PMID: 30675195 PMC6341771

[ref22] Tong L. Lan W. W. Lim R. R. Chaurasia S. S. (2014). S100A proteins as molecular targets in the ocular surface inflammatory diseases. Ocul. Surf. 12, 23–31. 10.1016/j.jtos.2013.10.001, PMID: 24439044

[ref23] Wang P. R. Xu M. Toffanin S. Li Y. Llovet J. M. Russell D. W. (2012). Induction of hepatocellular carcinoma by in vivo gene targeting. Proc. Natl. Acad. Sci. U. S. A. 109, 11264–11269. 10.1073/pnas.1117032109, PMID: 22733778 PMC3396480

[ref24] Wang L. Yan W. Li X. Liu Z. Tian T. Chen T.. (2019b). S100A10 silencing suppresses proliferation, migration and invasion of ovarian cancer cells and enhances sensitivity to carboplatin. J. Ovarian Res. 12:113. 10.1186/s13048-019-0592-3, PMID: 31739800 PMC6859630

[ref25] Wang C. Zhang C. Li X. Shen J. Xu Y. Shi H.. (2019a). CPT1A-mediated succinylation of S100A10 increases human gastric cancer invasion. J. Cell. Mol. Med. 23, 293–305. 10.1111/jcmm.13920, PMID: 30394687 PMC6307794

[ref26] Wu H. Tao J. Li X. Zhang T. Zhao L. Wang Y.. (2017). MicroRNA-206 prevents the pathogenesis of hepatocellular carcinoma by modulating expression of met proto-oncogene and cyclin-dependent kinase 6 in mice. Hepatology 66, 1952–1967. 10.1002/hep.29374, PMID: 28714063 PMC5696004

[ref27] Yammani R. R. (2012). S100 proteins in cartilage: role in arthritis. Biochim. Biophys. Acta 1822, 600–606. 10.1016/j.bbadis.2012.01.006, PMID: 22266138 PMC3294013

[ref28] Yang X. Popescu N. C. Zimonjic D. B. (2011). DLC1 interaction with S100A10 mediates inhibition of in vitro cell invasion and tumorigenicity of lung cancer cells through a RhoGAP-independent mechanism. Cancer Res. 71, 2916–2925. 10.1158/0008-5472.CAN-10-2158, PMID: 21372205 PMC3078213

[ref29] Ye X. Li C. Zu X. Lin M. Liu Q. Liu J.. (2019). A large-scale multicenter study validates aldo-keto reductase family 1 member B10 as a prevalent serum marker for detection of hepatocellular carcinoma. Hepatology 69, 2489–2501. 10.1002/hep.30519, PMID: 30672601 PMC6593451

[ref30] Zhang B. Dong L. W. Tan Y. X. Zhang J. Pan Y. F. Yang C.. (2013). Asparagine synthetase is an independent predictor of surgical survival and a potential therapeutic target in hepatocellular carcinoma. Br. J. Cancer 109, 14–23. 10.1038/bjc.2013.293, PMID: 23764751 PMC3708586

[ref31] Zhang Y. Ran Y. Xiong Y. Zhong Z. B. Wang Z. H. Fan X. L.. (2016). Effects of TMEM9 gene on cell progression in hepatocellular carcinoma by RNA interference. Oncol. Rep. 36, 299–305. 10.3892/or.2016.4821, PMID: 27220462

[ref32] Zhu A. X. Duda D. G. Sahani D. V. Jain R. K. (2011). HCC and angiogenesis: possible targets and future directions. Nat. Rev. Clin. Oncol. 8, 292–301. 10.1038/nrclinonc.2011.30, PMID: 21386818 PMC3266719

