# Supplementary figures and images for: S100 Calcium Binding Protein A10, A Novel Oncogene, Promotes the Proliferation, Invasion, and Migration of Hepatocellular Carcinoma
Source: Front Genet. 2021 Jun 11;12:695036. doi: 10.3389/fgene.2021.695036 (PMC8226228; doi:10.3389/fgene.2021.695036)

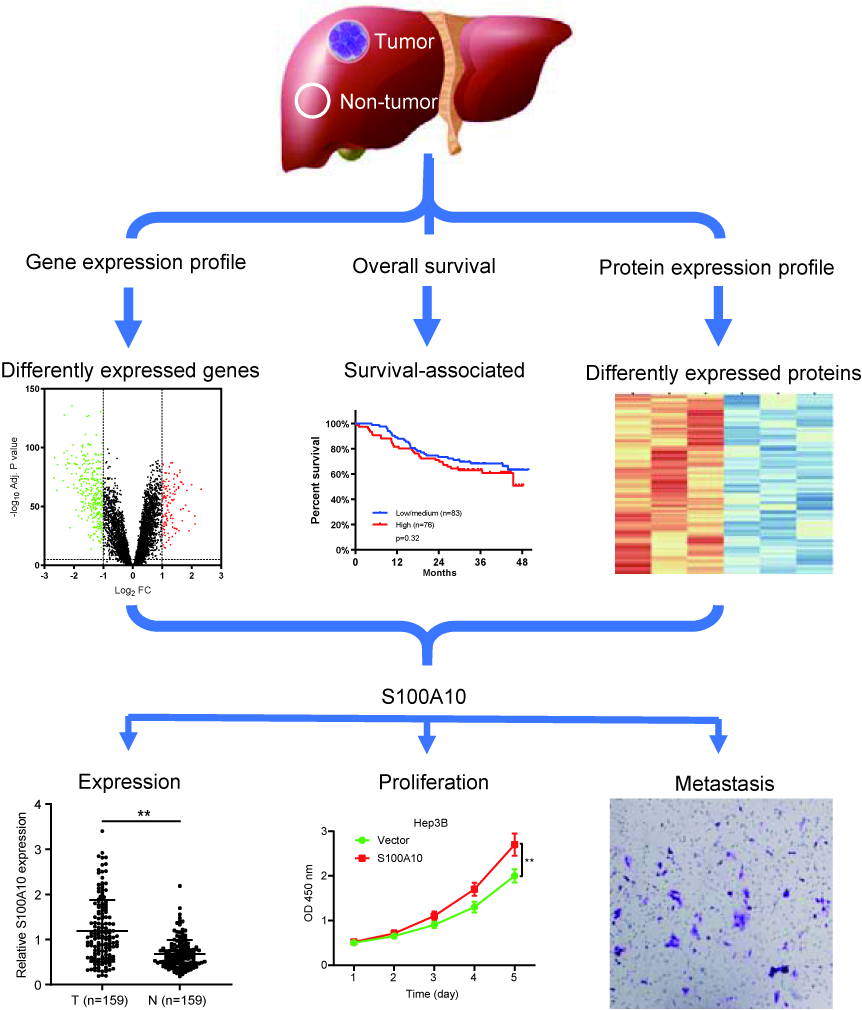

Supplement: Supplementary Figure 1 — The survival-associated DEGs were identified as potential oncogenes for further validation. [file Image_1.TIF]

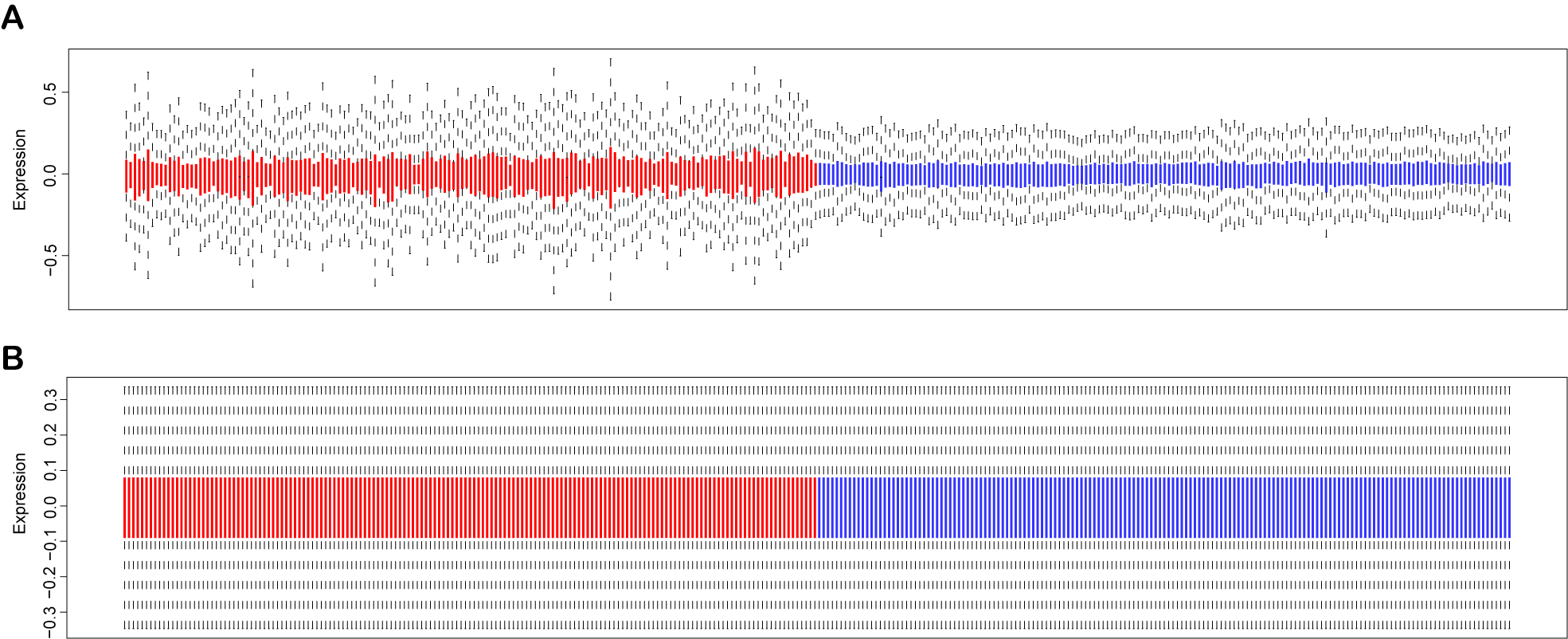

Supplement: Supplementary Figure 2 — The raw data from OEP000321 were normalized using the R package “limma.” [file Image_2.TIF]
